# A modified echocardiographic protocol with intrinsic plausibility control to determine intraventricular asynchrony based on TDI and TSI

**DOI:** 10.1186/1476-7120-7-46

**Published:** 2009-09-25

**Authors:** Henryk Dreger, Adrian C Borges, Bruno Ismer, Sebastian Schattke, Berthold Stegemann, Gert Baumann, Christoph Melzer

**Affiliations:** 1 Medizinische Klinik für Kardiologie und Angiologie, Campus Mitte, Charité - Universitätsmedizin Berlin, Germany; 2 Klinik für Innere Medizin, Universität Rostock, Germany; 3 Biotronik GmbH & Co. KG, Berlin, Germany

## Abstract

**Background:**

Established methods to determine asynchrony suffer from high intra- and interobserver variability and failed to improve patient selection for cardiac resynchronization therapy (CRT). Thus, there is a need for easy and robust approaches to reliably assess cardiac asynchrony.

**Methods and Results:**

We performed echocardiography in 100 healthy subjects and 33 patients with left bundle branch block (LBBB). To detect intraventricular asynchrony, we combined two established methods, i.e., tissue synchronization imaging (TSI) and tissue Doppler imaging (TDI). The time intervals from the onset of aortic valve opening (AVO) to the peak systolic velocity (S') were measured separately in six basal segments in the apical four-, two-, and three-chamber view. Color-coded TSI served as an intrinsic plausibility control and helped to identify the correct S' measuring point in the TDI curves. Next, we identified the segment with the shortest AVO-S' interval. Since this segment most likely represents vital and intact myocardium it served as a reference for other segments. Segments were considered asynchronous when the delay between the segment in question and the reference segment was above the upper limit of normal delays derived from the control population. Intra- and interobserver variability were 7.0% and 7.7%, respectively.

**Conclusion:**

Our results suggest that combination of TDI and TSI with intrinsic plausibility control improves intra- and interobserver variability and allows easy and reliable assessment of cardiac asynchrony.

## Background

In a significant percentage of patients with chronic heart failure (CHF), left ventricular (LV) dysfunction is accompanied by conduction disorders such as left bundle branch block (LBBB) [1]. This frequently leads to intraventricular asynchrony which impairs LV contraction, aggravates mitral regurgitation and consequently further promotes LV remodeling. Interestingly, however, several reports suggest that QRS duration is not correlated with mechanical intraventricular asynchrony [1,2]. Accordingly, multiple smaller, mostly single-center studies proposed various echocardiographic approaches for the detection of relevant intraventricular asynchrony [1]. Bax *et al*. suggested measurement of the maximal delay between peak systolic velocities (Ts-peak) in four basal segments. In 85 patients, this parameter predicted reverse LV remodeling after cardiac resynchronization therapy (CRT) with a sensitivity and specificity of 92% [3]. Another promising approach is based on the standard deviations of the times from QRS to peak systolic velocity in twelve LV segments (Ts-SD-12). In two smaller studies, Yu *et al*. identified Ts-SD-12 to be an effective independent predictor of reverse remodeling with a sensitivity of 96% and a specificity of 78% [4,5]. However, the PROSPECT trial - the largest multi-center study published to date - failed to confirm the reliability and effectiveness of twelve analyzed echocardiographic asynchrony measures - including Ts-SD-12 and a Ts-peak variant - to predict response to CRT [6]. A major concern with currently used asynchrony measures lies in their high intra- and interobserver variability which for one tested parameter (i.e., the septal-posterior wall motion delay) was as high as 24.3% and 72.1%, respectively, in the PROSPECT trial [6]. Even more robust approaches such as Ts-SD-12 and a modified Ts-peak measurement (in six basal segments) had an interobserver variability of 33.7% and 31.9%, respectively, and are thus insufficient for reliable assessment of asynchrony in the clinical routine [6]. Accordingly, current CRT guidelines do not recommend echocardiographic assessment of mechanical asynchrony [7]. Several studies, however, suggest that placing the left ventricular lead close to the most delayed segment might improve response to CRT [8-13]. As this requires a reliable identification of asynchronous segments, improvement of echocardiographic parameters for cardiac asynchrony remains clinically important.

### Aim

In order to both improve and simplify echocardiographic assessment of asynchrony, we sought to address the problem of intra- and interobserver variability by combination of two established echocardiographic methods - tissue Doppler imaging (TDI) [14] and tissue synchronization imaging (TSI) [15,16]. The aim of the present study was to define the upper limits of normal delays between six basal LV segments by examination of 100 healthy subjects. We then examined 33 LBBB patients and sought to identify asynchronous segments by using a new algorithm and the upper limits of normal derived from the control population.

## Methods

### Study population

We performed echocardiography in 100 healthy subjects and 33 patients with left bundle branch block (LBBB, QRS duration ≥ 120 ms) using a Vivid 7 ultrasound system (GE Medical Systems, Horton, Norway). LBBB patients were examined by echocardiography in our center for symptoms of heart failure or as a follow-up after myocardial infarction. Table 1 gives further information on the study population. The study conforms with local university ethics guidelines and the principles outlined in the Declaration of Helsinki.

**Table 1:** Characteristics of the study population.

	**control**	**LBBB**
n	100	33
males, n (%)	64 (64.0%)	23 (69.7%)
age, years	52.5 ± 16.9	69.7 ± 9.1
QRS, ms	82.4 ± 4.2	149.7 ± 15.5
LVEF, %	59.7 ± 2.1	34.6 ± 12.3
heart rate, min^-1^	72.0 ± 10.8	69.3 ± 11.3
cardiomyopathy		
ischemic, n (%)	-	17 (51.5%)
dilated, n (%)	-	10 (30.3%)
hypertensive, n (%)	-	2 (6.1%)
valvular, n (%)	-	1 (3.0%)

Values are mean ± SD when appropriate.

### Measurement of AVO-S' intervals in six basal segments

**Figure 1:**
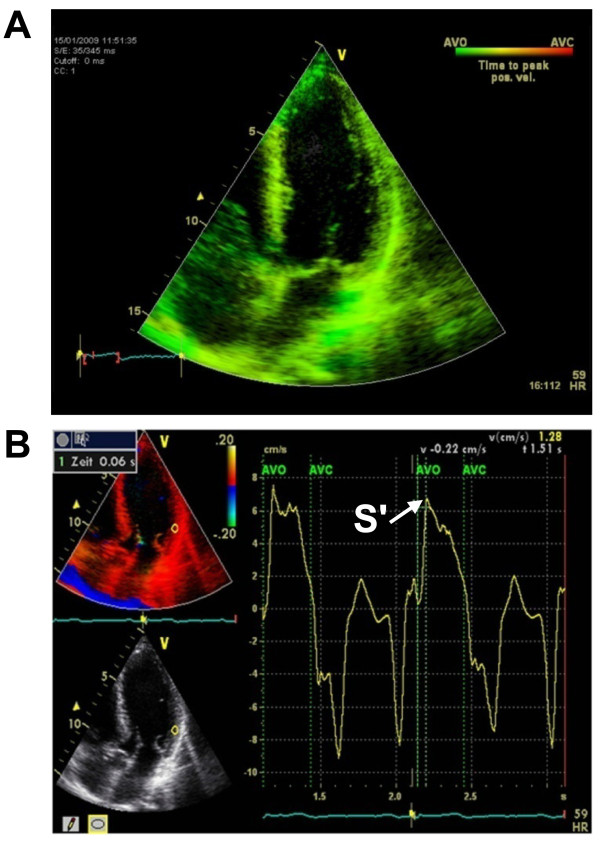
**A) TSI in the apical four-chamber view**. All LV segments are color-coded in green. Therefore, S' must be within the first third of the transaortal flow in the following TDI measurements. B) TDI in the apical four-chamber view. The interval between the aortic valve opening (AVO) and the peak systolic velocity (S') is 60 ms. In agreement with the TSI data, S' can be found within the first third of the transaortal flow.

As a first step, we determined the onset of the aortic valve opening (AVO) as a temporal reference point for all subsequent measurements by pulsed wave Doppler in the five-chamber view. Secondly, we performed TSI (triggered by the AVO) in the four-, two- and three-chamber views. TSI automatically color-codes time-to-peak tissue Doppler velocities, with colors ranging from green (at the beginning of the transaortal flow) through yellow and orange to red (at the end of the transaortal flow) (Figure 1A). Since TSI is not capable to quantify delays it only served as a qualitative analysis of LV synchrony. We then measured the intervals between the AVO and the peak systolic velocity (S') by TDI with a time resolution of 10 ms in six basal segments - i.e., in the septal and lateral segments (four-chamber view), in the anterior and inferior segments (two-chamber view) and in the anteroseptal and posterior segments (three-chamber view) (Figure 1B). Qualitative timing of the peak systolic velocity obtained from color-coded TSI was then compared to the AVO-S' intervals measured by TDI. Matching TDI and TSI served as an intrinsic plausibility control and was used to confirm correct determination of AVO-S' intervals by TDI. To further illustrate our approach, we prepared a sample clip of a control subject without asynchrony [see Additional file 1] as well as a sample clip of an LBBB patient with septal and inferior asynchrony [see Additional file 2].

### Calculation of upper limits of normal delays

Using the AVO-S' intervals, we calculated the delays (i.e., the AVO-S' differences or, in other words, the differences in the time each segment needed to reach peak systolic velocity) between all analyzed segments in the control population (Table 2). The upper limits of normal delays were defined as the mean plus two standard deviations (SD) of the delays from the control population (Table 3).

**Table 2:** Mean delays between the indicated basal LV segments in the control population.

	**septal**	**anteroseptal**	**anterior**	**lateral**	**posterior**	**inferior**
septal	--	9.3 ± 8.5	12.5 ± 13.3	12.3 ± 11.6	12.6 ± 14.1	14.1 ± 14.0
anteroseptal	9.3 ± 8.5	--	11.0 ± 14.3	11.4 ± 11.0	11.7 ± 13.0	12.0 ± 12.7
anterior	12.5 ± 13.3	11.0 ± 14.3	--	12.2 ± 13.1	11.9 ± 13.5	12.0 ± 14.1
lateral	12.3 ± 11.6	11.4 ± 11.0	12.2 ± 13.1	--	9.5 ± 8.2	8.6 ± 9.5
posterior	12.6 ± 14.1	11.7 ± 13.0	11.9 ± 13.5	9.5 ± 8.2	--	8.7 ± 9.2
inferior	14.1 ± 14.0	12.0 ± 12.7	12.0 ± 14.1	8.6 ± 9.5	8.7 ± 9.2	--

Values are mean ± SD [ms].

**Table 3:** Upper limits of normal delays (in ms) between the indicated basal LV segments derived from the control population.

	**septal**	**anteroseptal**	**anterior**	**lateral**	**posterior**	**inferior**
septal	--	26	39	35	41	42
anteroseptal	26	--	40	33	38	37
anterior	39	40	--	38	39	40
lateral	35	33	38	--	26	28
posterior	41	38	39	26	--	27
inferior	42	37	40	28	27	--

Calculated by .

### Identification of asynchronous regions

To detect asynchronous regions, we first identified the segment with the shortest AVO-S' interval. As this segment most likely represents vital and intact myocardium it served as a reference for other segments. We then calculated the delays between all other segments and this reference segment. Segments were considered asynchronous when the delay between the segment in question and the reference segment was above the upper limit of normal delays derived from the control population.

### Intra- and interobserver variability

For the analysis of intra- and interobserver variability, ten randomly chosen healthy subjects were reexamined by the original and a second investigator. Variability was analyzed by calculation of adjusted coefficients of variation (CV) defined as the ratio of the standard deviation (of the differences between the repeated measurements) and the mean of the absolute AVO-S' values.

### Statistics

Statistical significance was calculated by Mann-Whitney Rank Sum and z-tests when appropriate (SigmaStat 3.0, SPSS, Inc.). An error probability of *p *< 0.05 was regarded as significant. Receiver operating characteristic (ROC) curves were calculated using SPSS 17.0 (SPSS, Inc.).

## Results

The delays between all analyzed segments were markedly longer in LBBB patients as compared to the healthy controls (Table 4). The prevalence of asynchronous segments - according to calculated upper limits of normal delays derived from the control population (Table 3) - is given in Table 5. Except for the anterior wall, asynchrony was significantly more prevalent in all other segments in LBBB patients compared to healthy controls. 82% of LBBB patients had more than one asynchronous segment. In contrast, 9% of the control population had more than one AVO-S' interval above the upper limit of normal (Table 6).

**Table 4:** Mean delays between the indicated basal LV segments in LBBB patients.

**mean delay**	**septal**	**anteroseptal**	**anterior**	**lateral**	**posterior**	**inferior**
septal	--	36.3 ± 33.3	52.6 ± 35.7	65.2 ± 27.3	58.1 ± 30.8	45.6 ± 28.6
anteroseptal	36.3 ± 33.3	--	25.2 ± 33.9	48.1 ± 35.7	46.9 ± 36.1	52.5 ± 35.1
anterior	52.6 ± 35.7	25.2 ± 33.9	--	38.1 ± 37.0	41.9 ± 36.9	50.3 ± 36.6
lateral	65.2 ± 27.3	48.1 ± 35.7	38.1 ± 37.0	--	23.8 ± 23.7	32.5 ± 31.8
posterior	58.1 ± 30.8	46.9 ± 36.1	41.9 ± 36.9	23.8 ± 23.7	--	22.5 ± 25.6
inferior	45.6 ± 28.6	52.5 ± 35.1	50.3 ± 36.6	32.5 ± 31.8	22.5 ± 25.6	--

Values are mean ± SD [ms].

**Table 5:** Prevalence of asynchronous segments.

	**control**	**LBBB**	***p *value**
septal, n (%)	7 (7%)	24 (73%)	< 0.001
anteroseptal, n (%)	6 (6%)	11 (33%)	< 0.001
anterior, n (%)	2 (2%)	2 (6%)	0.551
lateral, n (%)	4 (4%)	10 (30%)	< 0.001
posterior, n (%)	5 (5%)	14 (42%)	< 0.001
inferior, n (%)	8 (8%)	17 (52%)	< 0.001

Segments were considered asynchronous when the delay between the segment in question and the reference segment (i.e., the segment with the shortest AVO-S' interval) was above the upper limit of normal (Table 3).

**Table 6:** Number of asynchronous segments per subject.

**Number of asynchronous segments**	**control**	**LBBB**	***p *value**
0	82 (82%)	0 (0%)	< 0.001
1	9 (9%)	6 (18%)	0.271
2	6 (6%)	12 (36%)	< 0.001
3	2 (2%)	13 (39%)	< 0.001
4	0 (0%)	1 (3%)	0.568
5	1 (1%)	1 (3%)	0.995
> 0	18 (18%)	33 (100%)	< 0.001

All segments averaged, intra- and interobserver variability was 7.0% and 7.7%, respectively (Table 7).

**Table 7:** Intra- and interobserver variability presented as coefficients of variation.

	**intraobserver**	**interobserver**
septal	6.0%	6.0%
anteroseptal	7.4%	7.9%
anterior	7.1%	7.1%
lateral	8.3%	9.1%
posterior	5.5%	9.1%
inferior	7.7%	6.7%
mean	7.0%	7.7%

**Figure 2:**
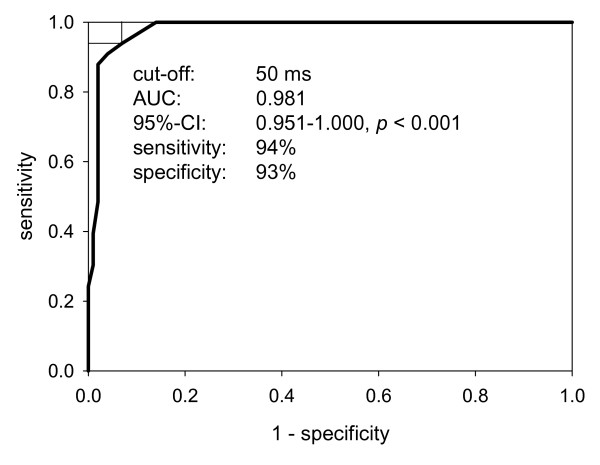
**Receiver operating characteristic curve analysis was used to further evaluate the longest intraventricular delay, i.e., the delay between the segments with the shortest and longest AVO-S' interval**. A cut-off of 50 ms had a sensitivity of 94% and specificity of 93% to discriminate control and LBBB patients.

To evaluate our protocol, we calculated ROC curves for the ability of the longest intraventricular delay - i.e., the delay between the segments with the shortest and the longest AVO-S' interval - to discriminate control subjects from LBBB patients. As depicted in Figure 2, a cut-off value of 50 ms had a sensitivity of 94% and a specificity of 93% to correctly identify LBBB patients.

## Discussion

Cardiac resynchronization (CRT) therapy aims at reverse remodeling and improvement of hemodynamics by amelioration of asynchronous LV contraction. Since all tested echocardiographic parameters failed to reliably predict a positive response to CRT in the PROSPECT trial, echocardiographic assessment of cardiac asynchrony has no evidence-based role in patient selection for CRT and, accordingly, is not recommended by current guidelines [7]. While decision for CRT is now routinely based on the QRS duration, electrical asynchrony is not always associated with mechanical asynchrony [1]. As the latter represents the basis of the pathophysiological concept of CRT, we believe it would be reasonable to screen patients for evidence of mechanical asynchrony. In addition, several studies suggest that response to CRT can be improved by placing the LV lead near the most delayed region [8-13]. These results call for new echocardiographic approaches which not only discriminate patients with cardiac asynchrony but also identify the most delayed myocardial segments. This requirement is further emphasized by our data which clearly demonstrate that the localization of asynchronous segments is subject to a high interindividual variance (Table 5).

In our opinion, a major problem of the parameters analyzed by the PROSPECT trial lies in their high intra- and interobserver variability which makes them too inaccurate for the clinical routine. Thus, the aim of our study was to establish a new, more reliable and straightforward protocol to determine intraventricular asynchrony based on two established methods. Indeed, reexamination of a part of our study population revealed that combination of both TSI and TDI reduced the intra- and interobserver variability to 7.0% and 7.7%, respectively (Table 7).

Assessment of cardiac asynchrony by the Ts-SD-12 index as proposed by Yu *et al*. is either laborious or based on TSI data obtained by a 3D transducer [4,5]. In our experience, this approach requires an optimal acoustic window - which is a condition frequently not given in the clinical routine. In contrast, measuring TSI separately in the apical four-, three- and two-chamber view using a 2D probe is feasible in most patients. As it is comparatively examiner-independent, automatic color-coding of the time-to-peak tissue Doppler velocities is a major advantage of TSI. Unfortunately, however, TSI allows only qualitative - and not quantitative - analysis of cardiac asynchrony. Therefore, our approach is based on combination of both TSI and TDI data.

So far, TDI is mainly used to identify asynchrony in septal and lateral segments which consequently does not yield information on the most delayed segment [14,15]. In our opinion, it appears obvious that screening for asynchrony should include as many segments as possible.

**Figure 3:**
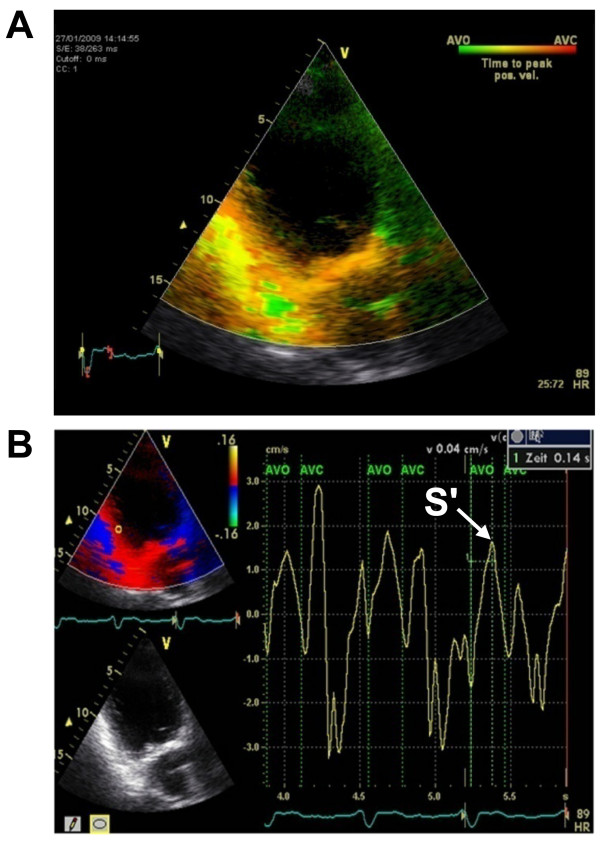
**Example of a patient with a reduced acoustic window**. A) TSI in the apical two-chamber view. The inferior segment is color-coded in orange. Therefore, S' must be within the middle third of the transaortal flow. This information serves as an intrinsic plausibility control and helps to identify the correct S' in the subsequent TDI measurements - especially under impaired ultrasound conditions as depicted in here. B) TDI in the apical two-chamber view. The interval between the aortic valve opening (AVO) and the peak systolic velocity (S') is 140 ms. In agreement with the TSI data, S' can be found within the middle third of the transaortal flow.

In our experience, identification of the correct S' is often difficult in the TDI curve. To address this possible source of error, we used the aortic valve opening as a reference point. In addition, we measured each segment separately thereby eliminating the need to superimpose several curves. Furthermore, using TSI data as an intrinsic plausibility control frequently helps to identify the correct S'. For example, if the segment in question is color-coded in orange, the peak velocity must be within the middle third of the transaortic flow in the TDI curve (Figure 3).

Numerous previous studies have separately demonstrated the usefulness of both TDI [3-5,14,17,18] and TSI [11,16,19,20]. Combining TDI and TSI to predict response to CRT, however, has only been proposed before by Gorcsan *et al*. [15]. Notably, though, the authors used TSI mainly as guidance to place the TDI regions of interest and, furthermore, identified asynchronous segments only by calculating the difference in time-to-peak velocity of opposing walls. In contrast, our protocol employs a strict combination of both TDI and TSI performed in six basal segments.

While our approach is based on two established methods, we here introduce a novel protocol to determine asynchronous segments by calculating intraventricular delays between all basal segments and a defined reference segment. In order to evaluate the plausibility of our data, we calculated the longest intraventricular delay, i.e., the delay between the segments with the shortest and the longest AVO-S' interval, for each subject. A maximum delay cut-off of 50 ms had a sensitivity of 94% and a specificity of 93% to discriminate control subjects and LBBB patients (Figure 2). This is in agreement with the pathophysiological model of cardiac asynchrony and thus suggests that our approach to calculate intraventricular delays is feasible and valid.

### Advantages

A major advantage of our approach lies in its intrinsic plausibility control due to strict combination of two validated asynchrony measures which - according to our data - reduces the intra- and interobserver variability. Since both TDI and TSI are robust and relatively easy to learn our approach is not restricted to experienced examiners and patients with an optimal acoustic window. Furthermore, examination is not time-consuming and can be performed in about ten minutes. Notably, our method allows a precise localization of the most delayed segment which might help improve left ventricular lead placement.

### Limitations

The present study is somewhat limited by the heterogeneity of our two study groups - especially regarding the left ventricular ejection fraction (LVEF) which on average was considerably lower in LBBB patients. However, the upper limits of normal that were used to determine asynchronous segments were derived entirely from our control population - independent from our patient group. Thus, their validity may be tested in follow-up studies which include other patient populations, e.g., patients with normal LVEF and LBBB or patients with reduced LVEF in the absence of conduction disorders.

Our study is further limited by the lack of data on CRT patients. Based on the encouraging results of this pilot study, however, we are currently applying our approach in CRT patients.

Moreover, our approach appears to be oversensitive as all LBBB patients had at least one asynchronous segment. On the other hand, this suggests that our approach would not deny treatment to LBBB patients eligible for CRT according to current guidelines. It may, however, help to improve response to CRT by guiding LV lead placement and by identifying patients with mechanical asynchrony and narrow QRS complexes.

## Conclusion

In summary, our approach to assess cardiac asynchrony aims at a practical combination of the advantages of TDI and TSI thereby eliminating possible sources of error. By examining 100 healthy subjects we were able to define upper limits of normal delays between all basal segments examinable from apical. Using these standard values we identified a prevalence of asynchronous segments in 33 LBBB patients which is comparable to numbers given in previous studies [13,16]. Due to the obligatory comparison of TDI and TSI data - which served as an intrinsic plausibility control - the intra- and interobserver variability was within an acceptable range.

Further experience in CRT patients will be necessary to evaluate whether our approach helps to improve assessment of cardiac asynchrony in the clinical routine. In addition to improving patient selection for CRT, our method might also be useful in the VV delay optimization.

## List of abbreviations

AVO: aortic valve opening; CHF: chronic heart failure; CRT: cardiac synchronization therapy; CV: coefficient of variation; LBBB: left bundle branch block; LV: left ventricle; LVEF: left ventricular ejection fraction; ROC: receiver operating characteristic; S': peak systolic velocity; SD: standard deviation; TDI: tissue Doppler imaging; TSI: tissue synchronization imaging.

## Competing interests

The authors declare that they have no competing interests.

## Authors' contributions

CM designed the study and performed the examinations. HD analyzed the data, performed the calculations and statistical analysis and wrote the manuscript. ACB analyzed and interpreted the data and critically revised the manuscript. SS reexamined control subjects to determine the interobserver variability. BI, BS and GB helped to analyze and interpret the data.

All authors have read and approved the final manuscript.

## Supplementary Material

Additional file 1 **Sample 1**. Sample clip of TSI and TDI measurements in a control patient without asynchrony. Click here for file

Additional file 2 **Sample 2**. Sample clip of TSI and TDI measurements in an LBBB patient with a reduced acoustic window and septal and inferior asynchrony. Click here for file
